# Aminopeptidase A initiates tumorigenesis and enhances tumor cell stemness via TWIST1 upregulation in colorectal cancer

**DOI:** 10.18632/oncotarget.15072

**Published:** 2017-02-03

**Authors:** Hui-Yu Chuang, Jeng-Kae Jiang, Muh-Hwa Yang, Hsei-Wei Wang, Ming-Chun Li, Chan-Yen Tsai, Yau-Yun Jhang, Jason C. Huang

**Affiliations:** ^1^ Department of Biotechnology and Laboratory Science in Medicine, National Yang-Ming University, Taipei, Taiwan; ^2^ Institution of Microbiology and Immunology, National Yang-Ming University, Taipei, Taiwan; ^3^ Department of Surgery, School of Medicine, National Yang-Ming University, Taipei, Taiwan; ^4^ Division of Colorectal Surgery, Department of Surgery, Taipei Veterans General Hospital, Taipei, Taiwan; ^5^ Institute of Clinical Medicine, National Yang-Ming University, Taipei, Taiwan; ^6^ Division of Hematology-Oncology, Department of Medicine, Taipei Veterans General Hospital, Taipei, Taiwan; ^7^ Institution of Biochemistry and Molecular Biology, National Yang-Ming University, Taipei, Taiwan; ^8^ Cancer Research Center, National Yang-Ming University, Taipei, Taiwan; ^9^ Department of Education and Research, Taipei City Hospital, Taipei, Taiwan; ^10^ Immunity and Inflammation Research Center, National Yang-Ming University, Taipei, Taiwan; ^11^ Genomic Research Center, Academia Sinica, Taipei, Taiwan; ^12^ Division of Pediatrics, Taipei City Hospital, Yang-Ming Branch, Taipei, Taiwan; ^13^ AIDS Prevention and Research Center, National Yang-Ming University, Taipei, Taiwan

**Keywords:** colorectal cancer, aminopeptidase A, metastasis, TWIST1, cancer stem cell

## Abstract

Metastasis accounts for the high mortality rate associated with colorectal cancer (CRC), but metastasis regulators are not fully understood. To identify a novel gene involved in tumor metastasis, we used oligonucleotide microarrays, transcriptome distance analyses, and machine learning algorithms to determine links between primary and metastatic colorectal cancers. Aminopeptidase A (APA; also known as *ENPEP*) was selected as our focus because its relationship with colorectal cancer requires clarification. Higher APA mRNA levels were observed in patients in advanced stages of cancer, suggesting a correlation between *ENPEP* and degree of malignancy. Our data also indicate that APA overexpression in CRC cells induced cell migration, invasion, anchorage-independent capability, and mesenchyme-like characteristics (e.g., EMT markers). We also observed TWIST induction in APA-overexpressing SW480 cells and TWIST down-regulation in HT29 cells knocked down with APA. Both APA silencing and impaired APA activity were found to reduce migratory capacity, cancer anchorage, stemness properties, and drug resistance *in vitro* and *in vivo*. We therefore suggest that APA enzymatic activity affects tumor initiation and cancer malignancy in a TWIST-dependent manner. Results from RT-qPCR and the immunohistochemical staining of specimens taken from CRC patients indicate a significant correlation between APA and TWIST. According to data from SurvExpress analyses of TWIST1 and APA mRNA expression profiles, high APA and TWIST expression are positively correlated with poor CRC prognosis. APA may act as a prognostic factor and/or therapeutic target for CRC metastasis and recurrence.

## INTRODUCTION

Colorectal cancer (CRC) is the second most diagnosed cancer in females and third in males worldwide. CRC incidence rates, which are rapidly increasing in many countries, are associated with risk factors such as unhealthy diet, smoking and obesity [1]. Studies have shown that nearly 43% of colon cancer patients developed liver metastases and 25% of CRC patients had both liver and lung metastases, with a five-year stage IV survival rate of less than 10% [2, 3]. Accordingly, the early detection of metastasis-associated genes may increase the lifespans of CRC patients.

Cancer-related epithelial-mesenchymal transition (EMT) is one of the most important mechanisms accounting for initial metastatic development [4]. Transdifferentiation is stimulated by cancerous microenvironments and triggered by transcription factors (TFs) such as SNAI1 (snail), SNAI2 (slug), the zinc-finger E-box-binding (ZEB) family, TWIST1 and TWIST2, among others [5]. EMT-related TFs are associated with cancer stemness properties [6]. Cancer cell mesenchymal phenotypes are linked with metastasis and stem-like properties. For example, circulating tumor cells (CTCs) express mesenchymal markers such as vimentin, TWIST and N-cadherin, and stem cell markers such as CD133^high^, ALDH1^high^ and CD44^high^/CD24^low^ [7, 8]. Combined, the evidence suggests that malignant cell invasion and dissemination occurs via EMT-mediated de-differentiation, with a change in self-renewal function to achieve colonization via re-differentiation at distant metastatic sites. Another factor in metastasis involves cancer stem cells (CSCs)—a minor population of malignant cancer cells with significant capabilities for self-renewal, differentiation, and therapy resistance that are strongly correlated with tumor recurrence [9]. Molecules that promote CSC generation in EMT or metastatic processes in CRC are still poorly understood.

Evidence from the high-throughput genomics process used for this study indicate the elevated expression of a unique gene, *ENPEP*, in metastatic colorectal carcinomas compared to *in situ* primary tumors. Glutamyl aminopeptidase—EC 3.4.11.7, also known as aminopeptidase A (APA), gp160, and CD249 [10]—is a type II transmembrane zinc metallopeptidase of the M1 family encoded by the *ENPEP* gene [11]. APA is abundantly expressed in the brush borders of the small intestine, renal glomeruli, and proximal renal tubules of early B-lineage cells collected from mice [12]. This ectopeptidase cleaves N-terminal glutamatic and aspartatic amino acid residues from polypeptide substrates such as angiotensin (Ang) II, cholecytokinin-8 (CCK8) *in vitro*, and neurokinin B [13, 14]. APA enzymatic activity is modulated by Ca^2+^ and brain renin–angiotensin system (RAS) homeostasis by converting Ang II to Ang III [15]. Several research teams have described APA involvement in different cancers—for example, promoting cell proliferation via the angiotensin II-AT_1_ axis in human prostate cancer [16] and in choriocarcinomas [17]. Others have described APA up-regulation in uterine cervix neoplastic lesions [18].

However, few efforts have been made to clarify the influence of APA on tumor progression mechanisms, or the functional role of APA in colorectal cancer. The primary motivations for the present study are understanding how APA performs its oncogenic role, and taking steps toward identifying one or more possible metastasis promotion mechanisms. The novel CRC metastasis-related gene that we identified may serve as a useful CRC diagnostic marker and/or therapeutic target.

## RESULTS

### Elevated expression of Aminopeptidase A (*ENPEP)* with CRC disease progression and cell mobility *in vitro*

**Figure 1:**
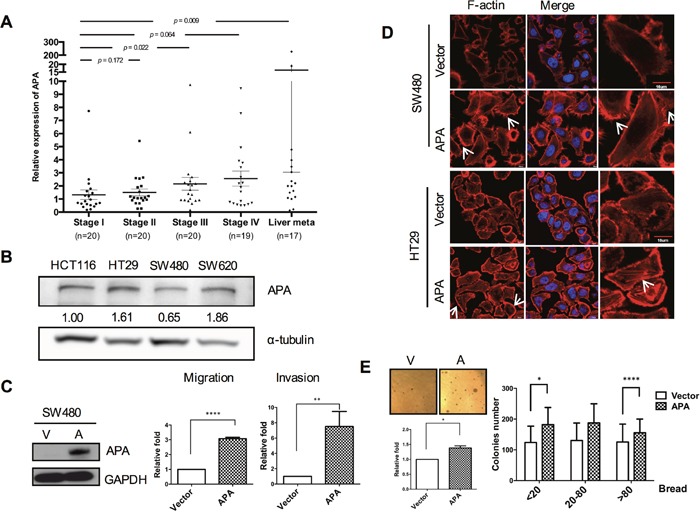
Aminopeptidase A expression increases with CRC progression and is associated with cell motility **A**. Graph plotting data from quantitative real-time RT-PCR analyses of ENPEP mRNA levels at different stages in CRC patients. Black lines indicate mean. *P <0.05; **P <0.01. **B**. Results from Western blot analyses of endogenous aminopeptidase A in four human CRC cell lines. **C**. Left, data from Western blot analyses of aminopeptidase A expression in pFlag-vector and pFlag-APA-transfected SW480 cells. Middle and right, migratory and invasive SW480 cell quantification. **D**. Immunofluorescence images of cell cytoskeletons following staining with rhodamine-phalloidin for F-actin in stable APA-overexpressed cells. Scale bars, 10 μm. **E**. Representative images of enhanced anchorage-independent growth capability of APA-overexpressed cells. Left, colony numbers were quantified using MetaMorph software with breadth parameters in ten randomly selected fields. Scale bars, 100 μm. Right, three breadth regions: 20, 20-80 and >80. *P <0.05; **P <0.01; ***P <0.001; ****P <0.001. Procedures were repeated a minimum of three times using duplicate wells for each sample. Student’s *t*-tests were used to determine statistical significance compared to a control group.

The *ENPEP* gene was selected from 157 genes with significantly different expression patterns in overlapping microarray data sets consisting of normal, tumorous and metastatic samples (Supplementary Figure 1a). Results from a gene ontology analysis to predict an APA biologic function tree indicate a link between APA and cell migration (Supplementary Figure 1b). We found that APA mRNA levels were significantly elevated in stage III and liver metastasis tissue samples compared to stage I samples (Figure 1A). According to these results, a significant correlation exists between APA expression and cancer progression. We used Western blotting to compare endogenous APA expression levels in four cell lines: SW480 (Dukes B), SW620 (Dukes C), HT29 (Dukes C) and HCT116 (Dukes D). Results indicate a correlation between APA protein levels and tumor grading sequences in SW480, SW620 and HT29 only (Figure 1B). This finding, which is consistent with our data for ENPEP mRNA levels in stage IV patients suggests that APA affects both Dukes B and Dukes C cells.

Due to their inclination toward lower APA expression, SW480 cells were chosen for APA overexpression. To assess the effects of APA overexpression on cell migration, we performed transient transfection and used Western blots to detect APA protein levels (Figure 1C, left). Results indicate increased migration and invasion activity in APA-overexpressing cells compared to vector control cells (Figure 1C, right). During cell motion, cells with high motility expressed actin or microtubule polymerization and lamellipodium protrusion [19]. This confirmed the effect of APA on the protrusive force of migration by enhancing actin filament polymerization at the leading edge. Stable APA-overexpressing SW480 and HT29 cell lines were established by lentiviral transduction (Supplementary Figure 1c) and subjected to immunofluorescence staining assays. As shown in Figure 1D, APA overexpression increased the formation of fluorescence-labeled filamentous F-actin, lamellipodia, and filopodia microspikes, thus contributing to elongated cell shapes. Next, we looked at the potential of APA overexpression to promote malignancy *in vitro*. Results from a soft agar analysis indicate (a) that the number of colonies increased in SW480 APA-overexpressing cells (Figure 1E, left), and (b) compared to a vector control, a colony breadth advantage exists in terms of anchorage-independent growth (Figure 1E, right). These data support our hypothesis regarding APA participation in CRC tumorigenesis and metastasis.

### Aminopeptidase A upregulates TWIST during cancer progression

**Figure 2:**
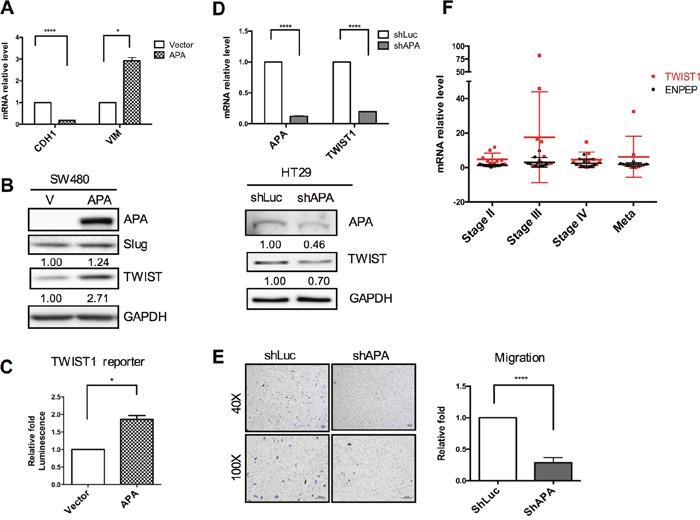
Positive correlation between APA and TWIST expression in CRC cell lines and patients **A**. EMT markers were validated in transfected APA-overexpressing cells as determined by qRT-PCR. pFlag-vector and pFlag-APA plasmids were transfected in SW480 cells for 24 h. Induced protein levels and TWIST reporter activity were analyzed by **B.** Western blots and **C.** luciferase reporter assays. *P <0.05 and ****P <0.001 (Student’s *t*-tests). **D**. Knocked down APA in HT29 cells via lentiviral shAPA infection. shLuc served as a control. mRNA levels and protein expression of APA and TWIST1 in APA-depleted HT29 cells were detected by qRT-PCR and Western blot assays, respectively. ****P <0.001 (Student’s *t*-tests). **E**. Images and data from cell migration analyses and quantification of APA-knockdown HT29 cells. Magnification: upper, 40x; lower, 100x. Scale bars, 100 μm. ****P <0.001 (Student’s *t*-tests). **F**. Quantitative real-time RT-PCR was used to compare APA and TWIST1 mRNA levels during different stages of paired clinical CRC samples. Results from a Pearson correlation test indicate a 0.638 correlation coefficient (*P*=0.159) between APA level and TWIST expression.

In cancer cells, the EMT process triggers an increase in invasive properties, as well as de-differentiation into a CSC-like phenotype via the complex regulation of key molecules such as Snail, Slug and TWIST [20]. As shown in Figure 2A, E-cadherin (CDH1) and vimentin (VIM) mRNA levels revealed a mesenchymal phenotype in SW480 APA-overexpressing cells compared to a vector control. Primary EMT regulator expression was determined by Western blots (Figure 2B). Results indicate significant upregulation of the TWIST but not the Slug protein during APA overexpression. We also observed a two-fold increase of TWIST promoter activity in APA-overexpressing SW480 cells compared to a vector control (Figure 2C).

Next, we looked at whether APA inhibition downregulated TWIST expression. In HT29 cells, we found that the APA knockdown process significantly reduced TWIST expression in terms of both mRNA and protein levels (Figure 2D), while concurrently attenuating cell motility (Figure 2E). Further, *TWIST1* mRNA increased as APA mRNA levels increased—a finding that was also applicable to our clinical CRC specimens (Figure 2F). Combined, these data suggest that APA is capable of upregulating TWIST expression in both CRC cell lines and clinical specimens, thus promoting tumor progression and motility.

### Aminopeptidase A-induced cancer cell stemness via TWIST regulation

**Figure 3:**
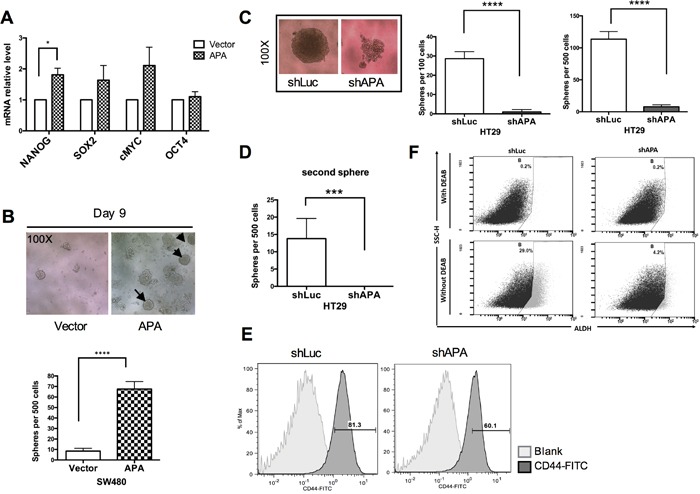
Stemness properties are enhanced in APA-overexpressed SW480 cells and impaired in APA-depleted HT29 cells **A**. Stemness gene expression in SW480 cells was validated following transfection with pFlag-vector and pFlag-APA plasmids. qRT-PCR was used to analyze mRNA expression (*P <0.05). **B**. Sphere formation analysis results for determining tumor initiation capability of APA-overexpressed cells. Upper, sphere-forming images from day 9. Lower, spheres from 500 SW480 cells, quantified after three weeks of incubation. Procedures were repeated a minimum of three times using ten wells for each sample. ****P <0.001. **C**. Left, sphere-forming images from 100 seeded and stable APA-depleted HT29 cells on day 14. Middle and right, quantitative sphere-forming results from 100 and 500 seeded cells on day 14. ****P <0.001. **D**. Self-renewal was analyzed using secondary sphere formation assays. Cells were passaged after 7 days of sphere formation; 100 cells from each group were re-seeded and spheres counted 14 days later. ***P <0.001. **E**. Flow cytometry was used to analyze CD44^+^ cells in APA-depleted or shLuc HT29 cells. **F**. ALDH activity was analyzed in the presence or absence of DEAB in APA-depleted or shLuc HT29 cells. Student’s *t*-tests were used to determine statistical significance compared to a control group.

TWIST has been shown to promote tumor initiation and cancer stem cell properties during the EMT process [6]. We therefore used qRT-PCR to analyze stemness and relative gene expression (Nanog, c-MYC, Oct-4 and Sox2) in both APA and vector-only transfectants (Figure 3A). Our data indicate that APA transfectants expressed a higher number of stemness genes. Inherent stemness traits can be enriched under serum-free spheroid culture conditions—the spheroids that are formed reflect the self-renewal capability of stem cells or cancerous stem-like cells. Observations during serum-free spheroid culturing indicate spheroid formation in APA-overexpressing cells starting on day 5 (Supplementary Figure 2a). Integral spheroids were clearly observed on day 9 (Figure 3B, upper), and spheroid numbers increased significantly in APA-overexpressing SW480 cells after three weeks (Figure 3B, lower). In contrast, APA knockdown in HT29 cells reduced their ability to form intact circular spheroids (Figure 3C, left and Supplementary Figure 2b). Further, the number of primary- and secondary-seeded spheroid cells in APA-knocked down HT29 cells decreased significantly (Figure 3C middle and right and Figure 3D). ALDH1 activity has been reported as a reliable index of CRC stem cells [21], and ALDH1 expression in CRC is strongly associated with and dependent on TWIST expression [22]. Our flow cytometry data show that CD44^+^ and ALDH^+^ cell populations significantly declined in APA-knocked down HT29 cells (Figure 3E and Figure 3F), suggesting that CSC-like characteristics are enhanced by APA expression.

### Aminopeptidase A mutation at metabolic activity site impairs cell mobility and CSC properties

**Figure 4:**
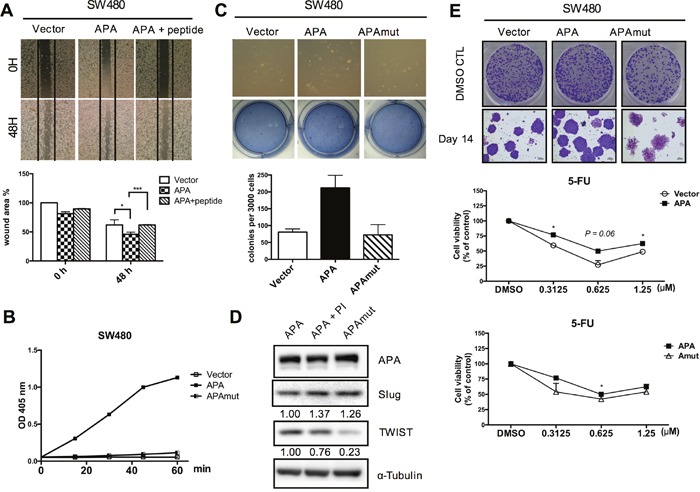
APA enzymatic activity is required for cancer cell migration and maintenance of cancer stemness in CRC cells **A**. Peptide inhibitor-treated APA-overexpressing cells were used to examine cell migration. Wound areas were quantified using ImageJ software. *P <0.05, ***P <0.001. **B**. APA enzymatic activity was determined in stably expressed vector, APA, and D221A mutant APA (APAmut) SW480 cells. **C**. Colony formation assays entailed 3,000 cells per well. Anchorage-independent growth effects were impaired in SW480-APAmut cells. *P <0.05. **D**. TWIST and Slug expression were detected by Western blotting. **E**. Representative clonogenic assay images showing the strong proliferation capability of overexpressed APA cells. 5-Fluoruracil (5-FU), a chemotherapy drug, was used to compare the drug-resistance capabilities of stable Vector, APA, and APAmut (D221A) SW480 cells at different dosages. *P <0.05. Student’s *t*-tests were used to determine statistical significance compared to a control group.

The CPRECESIC peptide specifically inhibits APA enzymatic activity [23]. In the present study we found that APA enzymatic activity was inhibited by treatment with 1 mM of the CPRECESIC peptide in SW480-APA cells (Supplementary Figure 3). Results from wound-healing assays indicate reduced cell motility following the inhibition of APA activity. A negative charge for aspartatic acid 221 is considered essential for the Ca^2+^ modulation of human APA enzymatic activity [24]. To determine whether the loss of APA activity exerted a negative effect on metastasis, we used site-directed mutagenesis to construct two human APA mutants with D221A substitutions: pFlag-APAmut and pLAS3w.Ppuro-APAmut. APA enzymatic activity in vector and SW480-APAmut cells were almost undetectable, even after 60 min (Figure 4B). The number of migratory cells decreased significantly in SW480-APAmut compared to SW480-APA cells and the vector control (Figure 4C).

We attempted to determine the importance of APA enzymatic activity for TWIST regulation and TWIST-induced phenotypes in cancer cells. Immunoblotting assay results show that the APA mutation significantly downregulated TWIST expression in SW480 cells (Figure 4D). We found that APA overexpression increased the number of cell colonies, but APA mutants formed smaller numbers of colonies in SW480 cells (Figure 4E, upper). Drug resistance (the primary reason for treatment failure) is characteristic of cancer stem-like cells [25]. Our data indicate that cells overexpressing APA but not APAmut showed greater resistance to 5-FU-induced growth inhibition compared to the vector control (Figure 4E, lower). Combined, these data suggest that APA peptidase activity is vital for APA-induced cell motility, cell proliferation, chemoresistance, and cancer stemness properties.

### APA-regulated TWIST expression via NF-κB translocation

**Figure 5:**
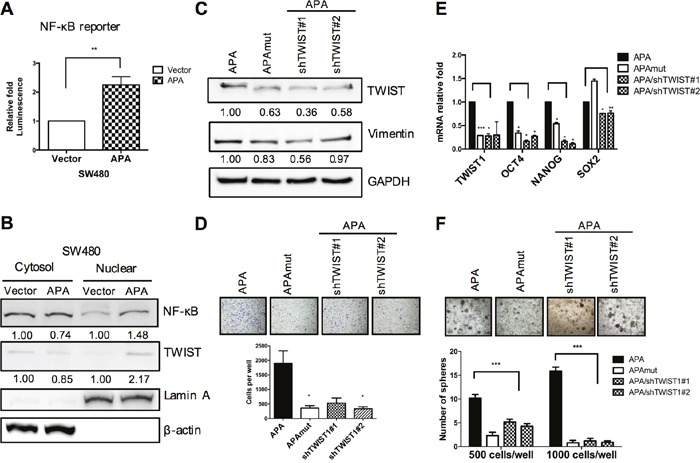
APA-mediated EMT and cancer stemness via NFκB-TWIST pathway activation in colon cancer **A**. SW480 cells were co-transfected with empty vector (pFlag-V) or APA (pFlag-APA), pNF-kB-luc, and pRL-SV40 plasmids for 48 hr. NF-kB reporter activity was measured using dual-luciferase reporter assays. **P <0.01. **B**. Nuclear and cytoplasmic fractions were separated from vector- or APA-expressing SW480 cells. NF-kB and TWIST translocation from cytosols into cell nuclei was detected by Western blotting. LaminA and β-actin served as loading controls for nuclear and cytoplasmic fractions, respectively. **C**. TWIST1 knockdown in APA-overexpressing SW480 cells (APA/shTWIST#1, APA/shTWIST#2), with TWIST and Vimentin expression detected by Western blotting. **D**. The migratory activity of TWIST1-depleted APA-overexpressing SW480 cells was measured by transwell migration assays. *P <0.05. **E**. Quantitative real-time RT-PCR analyses of TWIST1, OCT4, Nanog, and Sox-2 mRNA levels were performed following TWIST1 knockdown in APA-overexpressing SW480 cells. *P <0.05, **P <0.01, ***P <0.001. **F**. Sphere formation assays were performed in APA, APAmut, and TWIST1-depleted APA-overexpressing SW480 cells. ***P <0.001. Student’s *t*-tests were used to determine statistical significance compared to a control group.

To maintain EMT phenotype and cancer malignancy, the NF-κB pathway mediates several EMT-TFs, including Snail, Slug, TWIST, Sip1, ZEB1 and ZEB2 [26, 27]. In breast cancer, activated NF-κB p65 binds to a TWIST promoter and triggers EMT by inducing TWIST expression [28]. We therefore tried to determine whether a NFκB-TWIST1 signaling axis exists in APA-induced EMT, and found that NF-κB reporter activity was significantly upregulated in APA-overexpressed SW480 cells (Figure 5A). We also confirmed that APA induced NF-κB p65 translocation to the cell nucleus, and enhanced TWIST expression in nuclear fractions (Figure 5B). According to these results, TWIST regulation by APA is dependent on NF-κB signaling activation.

### TWIST knockdown in APA-overexpressing cells minimizes cell motion and CSC-like properties induced by enzymatic activity

To confirm that the effects of cell motility were mediated by TWIST due to APA overexpression, TWIST expression was knocked down in APA-overexpressing SW480 cells. Western blot results show that vimentin levels decreased in both APAmut and APA-/TWIST1 knockdown cells (APA/shTWIST1#1 and #2) (Figure 5C). Similar to APAmut-overexpressing cells, TWIST knockdown in APA-overexpressing SW480 cells significantly reduced cell migration ability (Figure 5D). Further, sphere formation assay results confirm that spheroid numbers decreased markedly following TWIST1 knockdown—a finding that is in agreement with reduced mRNA levels in stemness genes (Figure 5F and Figure 5E). Combined, the evidence indicates that APA-enhanced EMT and CSC-like properties were removed by the downregulation of TWIST expression.

### APA expression affects patient prognoses and tumorigenesis *in vivo*

**Figure 6:**
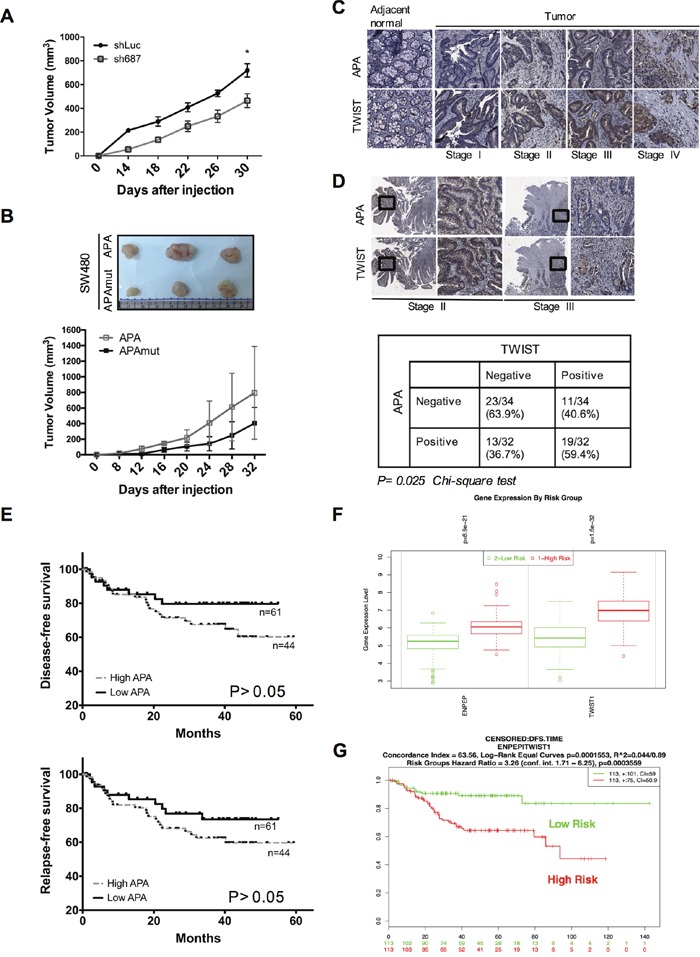
APA expression revealed enhanced tumorigenicity *in vivo*, and double-positive expression in APA/TWIST was correlated with poor prognoses in CRC patients **A**. APA-depleted or control HT29 cells or **B.** SW480 cells expressing APA or mutant APA were injected subcutaneously into nude mice (n=5 per group). Tumor volumes were measured every 4 days for 1 month. We used Student’s *t*-tests to compare the growth of pairs of tumors at each time point. *P <0.05. **C**. APA and TWIST expression was evaluated by immunohistochemical staining (40X) in a CRC tissue array (n=66, stages I to IV). **D**. Upper, representative staining images show a correlation between APA expression in stroma and the nuclear expression of TWIST in mucosa and submucosa (200X magnification within black frame). Lower, data from statistical analysis indicate a correlation between APA and TWIST expression. Statistical significance determined by Chi-square test. **E**. Kaplan–Meier survival analyses of disease-free survival (upper) and relapse-free survival (lower) were performed using samples collected from 105 CRC patients with high and low APA levels. Result from log-rank test indicate *P* = 0.157 on disease-free survival and *P* = 0.217 on relapse-free survival. Data from **F.** relative gene expression and **G.** Kaplan–Meier survival between APA (*ENPEP*) and TWIST (*TWIST1*) using SurvExpress are available at http://bioinformatica.mty.itesm.mx:8080/Biomatec/SurvivaX.jsp. Cutoff values were determined automatically using a colon cancer microarray data set (GSE14333) [52]. Result from log-rank test indicate *P <* 0.001 on disease-free survival.

To study the effects of APA on tumorigenicity, HT29-shLuc and HT29-shAPA cells were injected subcutaneously into nude mice. Data indicate that the tumorigenic capacity of APA knockdown cells decreased significantly compared to control cells (Figure 6A). Xenograft experiments were used to compare the effects of tumorigenesis in the presence or absence of APA enzymatic activity. As shown in Figure 6B, tumor growth decreased in the absence of APA activity. However, previous studies have shown that the APA amino acid sequence is 78% homologous to that of the murine BP-1/6C3 (APA) glycoprotein [29]. Mouse APA was overexpressed in murine colon adenocarcinoma CT26 cells and subcutaneously injected into BALB/c mice to examine the effects of APA on tumorigenesis. We observed that orthotopic CT26 tumor size increased noticeably in the APA-overexpressing group compared to the vector control group (Supplementary Figure 4).

According to these findings, APA expression and activity are important factors in CRC tumor formation. However, any clinical correlation between APA-plus-TWIST and patient outcomes remains unclear. To determine the prognostic significance of this potential correlation, we used IHC to analyze APA-TWIST co-expression in colorectal cancer samples, and found (a) significantly higher levels of APA and TWIST protein in tumor tissue compared to paired normal tissue (Figure 6C) and (b) large elevations of TWIST expression in nuclear bases associated with upregulated APA in tumor stroma (Figure 6D, upper). Results from double positive (59.4%) and double negative (63.9%) staining indicate a significant relationship between APA and TWIST (*P* <0.05), as calculated by a Pearson’s chi-squared analysis (Figure 6D, lower). We increased the number of patient specimens to 105 for a Kaplan–Meier survival analysis, and found that patients with higher levels of APA expression had significantly lower disease-free survival (DFS) and relapse-free survival (RFS) rates (Figure 6E). The multivariate analysis (Cox regression model) data indicate that metastasis was an independent significant prognostic factor for poor disease-free survival and association with APA expression (Table 1). Last, we used a SurvExpress online biomarker validation tool and microarray database to look for links between cancer gene expression and clinical outcomes, and to investigate DFS probabilities for patients with high levels of APA/TWIST expression. Results indicate a strong and positive link between APA/TWIST expression and cancer risk (Figure 6F). CRC patients experienced significant reductions in DFS with high APA/TWIST expression (Figure 6G). Combined, the IHC staining and microarray data suggest that APA expression induced increases in nuclear TWIST, and that APA is associated with poor clinical outcomes in colon cancer patients.

**Table 1:** Correlation of patient survival and APA expression and Clinicopathologic characteristics in the colorectal cancer cases

Variables	*P*	HR	95.0% CI for HR	
			lower	upper
Age				
≤71	0.534	1.705	0.759	3.832
>71				
Sex				
Male	0.698	1.173	0.524	2.628
Female				
TNM				
T1-T2	0.893	0.949	0.441	2.043
T3-T4				
N0	0.698	1.164	0.542	2.501
N1-N3				
M0	0.000	9.054	4.098	20.006
M1				

T, Primary Tumor; N, Regional Lymph Nodes; M, Distant Metastases; HR, hazard ratio

Data were analyzed using the Cox regression model test.

## DISCUSSION

To our knowledge, the present study is the first analysis of APA overexpression as a mediator for increased cell migration and cell stemness in colorectal cancer. Our data indicate an association between cancer promotion and APA enzymatic activity resulting in NF-κB activation and upregulated TWIST expression. Further, our clinical data show a positive correlation between APA expression and both metastasis and poor prognoses for colorectal cancer patients (Figures 1C, 6C and 6D). TWIST has been described as a CRC prognostic marker for overall survival (OS) in stage II but not stage III cases, and for DSF only in stage I cases [30]. However, our results suggest that CRC patient prognoses can be improved by measuring both APA and TWIST expression levels. Specifically, the data indicate that APA may serve as an early indicator of CRC progression—slightly upregulated during stage I, and significantly elevated during stage II.

One research team has reported links between *TWIST1* expression and both male hormones and advanced malignancies due to higher levels of *TWIST1* mRNA in males compared to females [31]. To confirm this gender link and to determine the regulating role of APA, we separated CRC specimens according to positive/negative APA and TWIST expression. Our data did not indicate any gender-related statistical differences (Supplementary Tables 1 and 2).

TWIST is a highly conserved basic helix-loop-helix (bHLH) transcription factor involved in biological development and linked to oncogenes [31]. Few efforts have been made to determine its malignant function, if any, in CRC. We observed significant increases in sphere formation (6-7 fold) in TWIST-upregulated cells induced by APA overexpression, but also found that knocked down TWIST in APA-overexpressing CRC cells eliminated this sphere formation capacity. According to our data, TWIST-induced stemness, which is also found in colon epithelial cells, can profoundly affect cancer stem cell populations.

TWIST, which is frequently overexpressed or hypermethylated, has important implications for tumor metastasis in colon cancer [31, 32]. It is therefore important to understand the role of upstream TWIST regulation in production and regulation pathways, and how it might provide benefits in terms of early cancer treatment [33]. Hypoxia-inducible factor 1α (HIF-1α) [34] and NF-κB [35] are dominant upstream TWIST inducers via their physical interaction. However, common colon cancer cell lines such as SW480 have KRAS mutations and NF-κB signaling cascades that are initiated by phosphati-dylinositol 3-kinase (PI3K)/Akt pathways during tumorigenesis [36]. We therefore focused on the NF-κB-TWIST axis to determine whether TWIST upregulation by APA occurs primarily via NF-κB activation. Phosphorylated AKT has been reported as promoting NF-κB activation and TWIST expression in hepatocellular carcinoma cells during EMT under hypoxic conditions [37]. The data indicate a direct link from PI3K/AKT to NF-κB and TWIST under such conditions. Hypoxia-inducible factor (HIF)-1α rapidly degrades due to proteasomes following ubiquitination by the pVHL complex in the presence of oxygen [38, 39]. At least one research team has demonstrated that APA is capable of regulating HIF-1α protein stability by partly blocking proteasomal degradation during ischemia-induced angiogenesis, and that APA expression also increases under hypoxic conditions [40]. However, HIF-1α expression in CRC patients is correlated with tumor growth, angiogenesis, metastasis, and VEGF expression [41]. In this study we failed to clarify the relationship between HIF-1α and APA. Additional effort is required to determine the mechanism underlying the role of HIF-1α in CRC APA overexpression.

In only one instance in the M1 family is enzymatic activity regulated by Ca^2+^: aminopeptidase [24]. We found that APA mutation D221A in SW480-overexpressing cells significantly impaired cancer malignancies both *in vivo* and *in vitro*. However, the catalytic activity of APA is dependent on a opened conformation which composed by the homodimer structure on head domain [42]. The conformational change was accompanied by positional changes of catalytic residues [43]. Increasing APA expression on the cell membrane may increase the catalytic activity of APA and metastasis-related substrates, such as angiotensin III.

One other aminopeptidase associated with tumor progression and metastasis is aminopeptidase N (APN/CD13), which is downstream of APA in the renin-angiotensin system. APN participates in angiogenesis, tumor cell invasion, and metastasis by induced extra-cellular matrix (ECM) degradation [44, 45]. Previous studies also suggesting that APN as an indicator of poor prognosis for lymph node metastasis in colon cancer [46]. An important direction for further work might be to study the oncogenic effects of APN in APA-overexpressing CRC samples. However, the conformation of catalytic cavity on mammalian APA are significantly narrower than APN, indicating that APN could recognize more substrates than APA but had poorer specificity. We suggest that substrates size of APA is relatively narrow, so that to develop specific inhibitors for APA activity may be better for metastatic colon cancer treated.

Further, specific APA binding motifs (e.g., the consensus sequence CPRECESIC) acted as inhibitors of APA activity, suppressing both VEGF-induced migration and endothelial cell proliferation [23]—results that are consistent with our findings for epithelial tumor cells. Monoclonal antibody treatment is currently considered a successful therapeutic strategy for both hematologic malignancies and solid tumors [47]. However, acute albuminuria and other forms of toxicity are linked to the targeting of the APA protein using the monoclonal antibodies ASD-37 and ASD-41 [48]. Accordingly, we are currently working on a CPRECESIC-based compound and a compound similar to the selective APA inhibitor (S)-3-amino-4-mercaptobutylsulphonic acid (EC33) [49], which specifically targets APA-induced CRC malignancies.

In conclusion, our results suggest a potential biomarker for use in early diagnosis, in metastasis-related prognosis, and as a CRC therapeutic target. It is possible that APA represents an upstream molecule that induces TWIST expression via NF-κB activation. The APA-induced NF-κB-TWIST pathway plays an important role in cell invasion, tumor initiation, and drug resistance, all of which are enzymatic activity-dependent. To date, no one has yet identified its APA substrates, which trigger downstream signaling associated with CRC tumorigenesis. Further studies are needed to clarify links between APA substrates and tumor progression signaling, which may provide clues for the development of drugs and other treatment modalities.

## MATERIALS AND METHODS

### Cell lines and transfection

SW480, HT29 and HCT116 human colon cancer cells were provided by Hsei-Wei Wang of National Yang-Ming University, Taiwan. SW620 cells were provided by the Resource Center of the National Research Program for Biopharmaceuticals (SB3, NSC 100-2325-B-080-001). Cell culturing is described in our Supplementary Materials and Methods section. Turbofect™ transfection reagent (Thermo Fisher Scientific, Waltham, MA, USA) was used for all transfection experiments according to manufacturer protocols.

### Microarray expression data sets

Data for a total of 253 publicly accessible array sets downloaded from Gene Expression Omnibus (GEO; http://www.ncbi.nlm.nih.gov/geo/) were used to determine relationships between colorectal carcinomas (CRC) and tumor metastasis. The sets include 17 for normal human tissues (4 Taiwanese colon samples plus 3 colon, 7 liver, and 3 lung tissue samples from GEO accession number GSE3526) and 236 colorectal adenocarcinomas (from the Expression Project for Ontology number GSE2109). In addition, 157 metastasis signature genes were collected from 162 patients (143 primary CRC and 19 metastasis); these were generated pre-2007 by the International Genomics Consortium [IGC]). Another set was collected from 78 patients (71 primary CRC patients and 7 metastasis) generated by the IGC in 2007, plus 2 paired Taiwanese primary and liver metastatic CRCs. To clarify the biological function of 157 genes identified in the Gene Ontology (GO) database (DAVID Bioinformatics Resources 2008 interface), we used a graph theory evidence-based method to agglomerate species-specific gene or protein identifiers.

### RNA extraction and real-time RT-PCR

RNA was extracted with a TRIzol reagent (Invitrogen, MA, USA) or RNA kit (Gene Mark, Taichung, Taiwan). Extracted RNA samples (2 μg) were used to synthesize cDNA with a RevertAid First Strand cDNA Synthesis Kit (Thermo Fisher Scientific). Quantitative real-time PCR was performed using SYBR Green MasterMix (Applied Biosystems, Carlsbad, CA, USA) and a StepOnePlus™ Real-Time PCR System (Thermo Fisher Scientific). Gene expression levels were normalized to human GAPDH and analyzed using the 2^−ΔΔCt^ method. Primers are shown in Supplementary Table 3. All tests were repeated a minimum of three times, with duplicate PCR reactions for each sample. For the experiment shown in Figure 3F, cDNA samples were randomly selected from a previous experiment (Figure 1A) to confirm relative TWIST1 mRNA levels.

### Plasmid construction and site-directed mutagenesis

Wild-type full-length human ENPEP cDNA clones were obtained from the Yang-Ming University VYM Genome Research Center (Taipei, Taiwan). The ENPEP gene was cloned into a pFLAG-CMV-2 vector (Sigma-Aldrich, MO, USA) for transient protein expression, and into a pLAS3w. Ppuro lentiviral transfer vector (National RNAi Core Facility, Academia Sinica, Taiwan) for stable cell production. Mouse ENPEP cDNA fragments were amplified from CT26 cell cDNA by PCR and cloned into pLAS3w.Ppuro lentiviral transfer vectors. Site-directed mutagenesis was used to alter the D221A amino acid of hAPA. See Supplementary Materials and Methods for details.

### Lentivirus production and infection

Lentivectors containing cDNA from the pLAS3w.Ppuro-vector control, pLAS3w.Ppuro-APA, pLAS3w.Ppuro-APAmut, pLAS3w.Pneo-vector control, and pLAS3w.Pneo-mAPA were overexpressed in CRC cells. For knockdown experiments, negative control lentivectors expressing shRNAs against human APA (shAPA), Twist1 (shTWIST#1, shTWIST#2), and luciferase (shLuc) were purchased from the National RNAi Core Facility, Taiwan. HEK 293T cells were used for lentivirus production according to protocols established by the National RNAi Core Facility (http://rnai.genmed.sinica.edu.tw). See Supplementary Materials and Methods for details.

### Anchorage-independent (soft-agar) colony formation assays

Soft agar mixtures (1.5 ml 0.5%; 2X Medium—L15, 10% FBS, 1% NEAA, 1% glutamine, 0.5% agar, 1% penicillin-streptomycin) were held in six-well plates for solidification. Next, 2 ml of 0.33% cell-agar mixture were added to the bottom layer and incubated for 2 weeks at 37°C with or without 5% CO_2_. Colonies were observed under a microscope; images were captured and analyzed by MetaMorph (Molecular Devices, CA, USA), or colonies were stained with Giemsa solution for quantification.

### Clonogenic assays

To determine long-term proliferation rates under different treatment conditions, 1 × 10^3^ CRC cells were seeded into six-well plates with 10% FBS-containing either DMSO or 5-FU (Sigma-Aldrich) at different dosages and held for an additional 14 days. Colonies were stained with 0.01% crystal violet and quantified using ImageJ software.

### Western blot analyses

Total cellular protein was extracted on ice using RIPA buffer containing a protease-phosphatase inhibitor cocktail (Sigma-Aldrich). Nuclear and cytoplasmic protein separation was performed using NE-PER™ Nuclear and Cytoplasmic Extraction Reagent kits (Thermo Fisher Scientific). Each protein sample (40 μg) was separated by SDS-PAGE and transferred onto PVDF membranes (Pall BioTrace, New York, USA) that were probed with antibodies as previously described [50]. Antibody details are given in Supplementary Table 4. Proteins were detected by enhanced chemiluminescence (PerkinElmer, Waltham, MA, USA) and quantified using ImageJ software.

### Cell migration and invasion assays

pFlag-vector and pFlag-APA-transfected cell migration and invasion assays were performed using transwell insert chambers in 24-well plates according to the manufacturer’s instructions (Costar, 8 μm pores; Corning, NY, USA). For invasion assays, the upper chambers of each insert were coated with 50 μl BD Matrigel Matrix (BD Biosciences, CA, USA). Inverted microscopy was used to count migratory and invasive cells in five representative fields for each insert.

### Wound healing assays

SW480-APA, shTWIST#1, and shTWIST#2 cells were seeded in 12-well plates to confluence. Cell monolayers were scratched with a 20 μl tip, followed by medium removal, washing, and the addition of fresh medium. Cells were cultured at 37°C for 48 h and photographed by inverted microscopy at 0, 24 and 48 h. Wound areas were measured using ImageJ.

### Immunofluorescence

SW480 and HT29 cells (3 × 10^5^ each) were seeded onto 12 mm glass coverslips and incubated for 24 h. Cells were fixed with 4% paraformaldehyde for 30 min, followed by three washes with PBS and blocking with 3% BSA (Sigma-Aldrich) at room temperature for 1 hour. Cells were incubated with rhodamine-phalloidin for 2 hours at RT. DAPI (1:10,000) was used for nuclear counterstaining. Slides were observed using confocal microscopy.

### Immunohistochemical analysis of human colon tumor tissue microarrays

CRC patient specimens were provided by Dr. J.K. Jiang following approval from the Institutional Review Board of Taipei Veterans General Hospital. The TNM staging system used for these analyses was from the American Joint Committee on Cancer (AJCC) [51]. IHC assays were used to analyze human BP1 and TWIST protein expression. The clinicopathologic characteristics of CRC patients that used in Figure 6E, Figure 6C and 6D were shown in Supplementary Table 5 and Table 6. For details, see Supplementary Materials and Methods.

### Luciferase reporter constructs and luciferase assays

Either the pXP2-TWIST1-luc or pNF-κB-luc reporter construct was used to determine TWIST1 or NF-κB promoter activity at the transcriptional level. Luciferase activity was analyzed with a dual-luciferase reporter assay system (Promega, Wisconsin, USA). For NF-κB luciferase activity measurements, SW480 cells (2 × 10^5^) were seeded in 24-well plates and transiently co-transfected with the pFLAG-vector control, or with pFLAG-APA (500 ng) and pNF-κB-luc (500 ng) plus pRL-SV40 (50 ng). For TWIST1 luciferase activity measurements, SW480 cells stably expressing the vector control or APA were co-transfected with pXP2-TWIST1-luc (1000 ng) and pRL-SV40 (100 ng). Cells were harvested at 24 and 48 hours. A Dual-Luciferase Reporter Assay System (Promega) was used to detect luciferase activity according to manufacturer protocols.

### ALDH activity assays

Aldehyde dehydrogenase activity was measured using ALDE-FLUOR kits (STEMCELL Technologies, Vancouver, BC, Canada) according to the manufacturer’s instructions. For details, see Supplementary Materials and Methods.

### APA enzymatic activity assays

APA enzymatic activity was measured spectrophotometrically as previously described [15]. Briefly, cells were trypsinized and adjusted to 1 × 10^6^ cells/ml in 1 ml 0.1 M Tris-HCl (pH 7.4)/2.5 mm CaCl_2_ with 1.5 mM α-L-glutamic acid-*p*-nitroanilide (Sigma-Aldrich). Following incubation at 37°C for 15, 30, 45 or 60 min, 200 μl substrate were added to a microcentrifuge tube with ice-cold PBS, centrifuged, and added with 200 μl supernatant to 96-well microtiter plates. APA enzymatic activity was measured at 405 nm.

### Sphere formation assays

Cells were adjusted to 100, 500 or 1,000 cells/200 μl suspended in sphere-forming medium consisting of serum-free DMEM/F-12 supplemented with 10 ng/ml human recombinant epidermal growth factor and 10 ng/ml basic fibroblast growth factor. Cells were cultured for 7-30 days prior to counting sphere numbers.

### Animal experiments

HT29-shLuc and HT29-shAPA cells (1 × 10^5^) were subcutaneously injected into BALB/cAnN.Cg-*Foxn1^nu^*/CrlNarl mice (6-8 weeks old). CT26-V and CT26-mAPA cells (5 × 10^4^) were subcutaneously injected into BALB/c mice (also 6-8 weeks old). Tumor volume was measured once every 4 days and calculated using the formula tumor volume (mm^3^) = (Length x Width^2^)/2.

### Statistical analyses

Data were analyzed using SPSS statistical software (v22.0). All results are reported as mean ± s.d. Student’s *t*-tests were performed to evaluate two independent experimental groups. For comparison of mRNA expression at different stages of colorectal cancer, the Mann-Whitney U-tests was performed because of samples did not indicate a normal distribution. The Pearson correlation tests were used to analyze correlations between two continuous factors. Overall survival and disease-free survival were analyzed by Kaplan-Meier method, and the log-rank test was applied to compare the cumulative survival durations in different patient groups. For comparison of correlation between expression of APA and TWIST on paired tissue, the Chi-square test was performed used. The multivariate Cox regression analysis were used to analyzed relationships between APA and independent effect of clinical pathological variables on survival. *P* < 0.05 was considered significant.

## SUPPLEMENTARY MATERIALS FIGURES AND TABLES







## Abbreviations

AngII: angiotensin II
APA: aminopeptidase A
bHLH: basic helix-loop-helix
CCK8: cholecytokinin-8
CRC: colorectal cancer
CSCs: cancer stem cells
CTCs: circulating tumor cells
DFS: disease-free survival (DFS)
EMT: epithelial-mesenchymal transition
GO: gene ontology
IHC: immunohistochemistry
mRNA: messenger RNA
RAS: renin-angiotensin system
RFS: relapse-free survival (RFS).

## References

[1] Jemal A, Bray F, Center MM, Ferlay J, Ward E, Forman D (2011). Global cancer statistics. CA Cancer J Clin.

[2] Jemal A, Siegel R, Ward E, Murray T, Xu J, Thun MJ (2007). Cancer statistics, 2007. CA Cancer J Clin.

[3] Urosevic J, Garcia-Albeniz X, Planet E, Real S, Cespedes MV, Guiu M, Fernandez E, Bellmunt A, Gawrzak S, Pavlovic M, Mangues R, Dolado I, Barriga FM (2014). Colon cancer cells colonize the lung from established liver metastases through p38 MAPK signalling and PTHLH. Nat Cell Biol.

[4] Javle MM, Gibbs JF, Iwata KK, Pak Y, Rutledge P, Yu J, Black JD, Tan D, Khoury T (2007). Epithelial-mesenchymal transition (EMT) and activated extracellular signal-regulated kinase (p-Erk) in surgically resected pancreatic cancer. Ann Surg Oncol.

[5] Puisieux A, Brabletz T, Caramel J (2014). Oncogenic roles of EMT-inducing transcription factors. Nat Cell Biol.

[6] Mani SA, Guo W, Liao MJ, Eaton EN, Ayyanan A, Zhou AY, Brooks M, Reinhard F, Zhang CC, Shipitsin M, Campbell LL, Polyak K, Brisken C (2008). The epithelial-mesenchymal transition generates cells with properties of stem cells. Cell.

[7] Theodoropoulos PA, Polioudaki H, Agelaki S, Kallergi G, Saridaki Z, Mavroudis D, Georgoulias V (2010). Circulating tumor cells with a putative stem cell phenotype in peripheral blood of patients with breast cancer. Cancer Lett.

[8] Iinuma H, Watanabe T, Mimori K, Adachi M, Hayashi N, Tamura J, Matsuda K, Fukushima R, Okinaga K, Sasako M, Mori M (2011). Clinical significance of circulating tumor cells, including cancer stem-like cells, in peripheral blood for recurrence and prognosis in patients with Dukes' stage B and C colorectal cancer. J Clin Oncol.

[9] Vermeulen L, Sprick MR, Kemper K, Stassi G, Medema JP (2008). Cancer stem cells--old concepts, new insights. Cell Death Differ.

[10] Nanus DM, Engelstein D, Gastl GA, Gluck L, Vidal MJ, Morrison M, Finstad CL, Bander NH, Albino AP (1993). Molecular cloning of the human kidney differentiation antigen gp160: human aminopeptidase A. Proc Natl Acad Sci U S A.

[11] Glenner GG, Mc MP, Folk JE (1962). A mammalian peptidase specific for the hydrolysis of N-terminal alpha-L-glutamyl and aspartyl residues. Nature.

[12] Li L, Wu Q, Wang J, Bucy RP, Cooper MD (1993). Widespread tissue distribution of aminopeptidase A, an evolutionarily conserved ectoenzyme recognized by the BP-1 antibody. Tissue Antigens.

[13] Migaud M, Durieux C, Viereck J, Soroca-Lucas E, Fournie-Zaluski MC, Roques BP (1996). The in vivo metabolism of cholecystokinin (CCK-8) is essentially ensured by aminopeptidase A. Peptides.

[14] Zini S, Fournie-Zaluski MC, Chauvel E, Roques BP, Corvol P, Llorens-Cortes C (1996). Identification of metabolic pathways of brain angiotensin II and III using specific aminopeptidase inhibitors: predominant role of angiotensin III in the control of vasopressin release. Proc Natl Acad Sci U S A.

[15] Rozenfeld R, Reaux A, Iturrioz X, Fassot C, Fournie-Zaluski MC, David C, Maigret B, Roques BP, Corvol P, Llorens-Cortes C (2003). Aminopeptidase A, generating one of the main effector peptides of the brain renin-angiotensin system, angiotensin III, plays a key role in central control of blood pressure. Proc West Pharmacol Soc.

[16] Teranishi J, Ishiguro H, Hoshino K, Noguchi K, Kubota Y, Uemura H (2008). Evaluation of role of angiotensin III and aminopeptidases in prostate cancer cells. Prostate.

[17] Ino K, Nagasaka T, Okamoto T, Uehara C, Nakazato H, Nakashima N, Mizutani S (2000). Expression of aminopeptidase A in human gestational choriocarcinoma cell lines and tissues. Placenta.

[18] Suganuma T, Ino K, Shibata K, Nomura S, Kajiyama H, Kikkawa F, Tsuruoka N, Mizutani S (2004). Regulation of aminopeptidase A expression in cervical carcinoma: role of tumor-stromal interaction and vascular endothelial growth factor. Lab Invest.

[19] Ridley AJ, Schwartz MA, Burridge K, Firtel RA, Ginsberg MH, Borisy G, Parsons JT, Horwitz AR (2003). Cell migration: integrating signals from front to back. Science.

[20] Sabbah M, Emami S, Redeuilh G, Julien S, Prevost G, Zimber A, Ouelaa R, Bracke M, De Wever O, Gespach C (2008). Molecular signature and therapeutic perspective of the epithelial-to-mesenchymal transitions in epithelial cancers. Drug Resist Updat.

[21] Huang EH, Hynes MJ, Zhang T, Ginestier C, Dontu G, Appelman H, Fields JZ, Wicha MS, Boman BM (2009). Aldehyde dehydrogenase 1 is a marker for normal and malignant human colonic stem cells (SC) and tracks SC overpopulation during colon tumorigenesis. Cancer Res.

[22] Kim YH, Kim G, Kwon CI, Kim JW, Park PW, Hahm KB (2014). TWIST1 and SNAI1 as markers of poor prognosis in human colorectal cancer are associated with the expression of ALDH1 and TGF-beta1. Oncol Rep.

[23] Marchio S, Lahdenranta J, Schlingemann RO, Valdembri D, Wesseling P, Arap MA, Hajitou A, Ozawa MG, Trepel M, Giordano RJ, Nanus DM, Dijkman HB, Oosterwijk E (2004). Aminopeptidase A is a functional target in angiogenic blood vessels. Cancer Cell.

[24] Goto Y, Hattori A, Mizutani S, Tsujimoto M (2007). Asparatic acid 221 is critical in the calcium-induced modulation of the enzymatic activity of human aminopeptidase A. J Biol Chem.

[25] Singh A, Settleman J. (2010). EMT, cancer stem cells and drug resistance: an emerging axis of evil in the war on cancer. Oncogene.

[26] Chua HL, Bhat-Nakshatri P, Clare SE, Morimiya A, Badve S, Nakshatri H (2007). NF-kappaB represses E-cadherin expression and enhances epithelial to mesenchymal transition of mammary epithelial cells: potential involvement of ZEB-1 and ZEB-2. Oncogene.

[27] Criswell TL, Arteaga CL (2007). Modulation of NFkappaB activity and E-cadherin by the type III transforming growth factor beta receptor regulates cell growth and motility. J Biol Chem.

[28] Li CW, Xia W, Huo L, Lim SO, Wu Y, Hsu JL, Chao CH, Yamaguchi H, Yang NK, Ding Q, Wang Y, Lai YJ, LaBaff AM (2012). Epithelial-mesenchymal transition induced by TNF-alpha requires NF-kappaB-mediated transcriptional upregulation of Twist1. Cancer Res.

[29] Lin Q, Taniuchi I, Kitamura D, Wang J, Kearney JF, Watanabe T, Cooper MD (1998). T and B cell development in BP-1/6C3/aminopeptidase A-deficient mice. J Immunol.

[30] Gomez I, Pena C, Herrera M, Munoz C, Larriba MJ, Garcia V, Dominguez G, Silva J, Rodriguez R, Garcia de Herreros A, Bonilla F, Garcia JM (2011). TWIST1 is expressed in colorectal carcinomas and predicts patient survival. PLoS One.

[31] Valdes-Mora F, Gomez del Pulgar T, Bandres E, Cejas P, Ramirez de Molina A, Perez-Palacios R, Gallego-Ortega D, Garcia-Cabezas MA, Casado E, Larrauri J, Nistal M, Gonzalez-Baron M, Garcia-Foncillas J (2009). TWIST1 overexpression is associated with nodal invasion and male sex in primary colorectal cancer. Ann Surg Oncol.

[32] Ruppenthal RD, Nicolini C, Filho AF, Meurer R, Damin AP, Rohe A, Alexandre CO, Damin DC (2011). TWIST1 promoter methylation in primary colorectal carcinoma. Pathol Oncol Res.

[33] Khan MA, Chen HC, Zhang D, Fu J (2013). Twist: a molecular target in cancer therapeutics. Tumour Biol.

[34] Yang MH, Wu KJ (2008). TWIST activation by hypoxia inducible factor-1 (HIF-1): implications in metastasis and development. Cell Cycle.

[35] Sosic D, Olson EN (2003). A new twist on twist--modulation of the NF-kappa B pathway. Cell Cycle.

[36] Bai D, Ueno L, Vogt PK (2009). Akt-mediated regulation of NFkappaB and the essentialness of NFkappaB for the oncogenicity of PI3K and Akt. Int J Cancer.

[37] Yan W, Fu Y, Tian D, Liao J, Liu M, Wang B, Xia L, Zhu Q, Luo M (2009). PI3 kinase/Akt signaling mediates epithelial-mesenchymal transition in hypoxic hepatocellular carcinoma cells. Biochem Biophys Res Commun.

[38] Salceda S, Caro J (1997). Hypoxia-inducible factor 1alpha (HIF-1alpha) protein is rapidly degraded by the ubiquitin-proteasome system under normoxic conditions. Its stabilization by hypoxia depends on redox-induced changes. J Biol Chem.

[39] Bruick RK, McKnight SL (2001). A conserved family of prolyl-4-hydroxylases that modify HIF. Science.

[40] Kubota R, Numaguchi Y, Ishii M, Niwa M, Okumura K, Naruse K, Murohara T (2010). Ischemia-induced angiogenesis is impaired in aminopeptidase A deficient mice via down-regulation of HIF-1alpha. Biochem Biophys Res Commun.

[41] Kuwai T, Kitadai Y, Tanaka S, Onogawa S, Matsutani N, Kaio E, Ito M, Chayama K (2003). Expression of hypoxia-inducible factor-1alpha is associated with tumor vascularization in human colorectal carcinoma. Int J Cancer.

[42] Yang Y, Liu C, Lin YL, Li F (2013). Structural insights into central hypertension regulation by human aminopeptidase A. J Biol Chem.

[43] Chen L, Lin YL, Peng G, Li F (2012). Structural basis for multifunctional roles of mammalian aminopeptidase N. Proc Natl Acad Sci U S A.

[44] Pasqualini R, Koivunen E, Kain R, Lahdenranta J, Sakamoto M, Stryhn A, Ashmun RA, Shapiro LH, Arap W, Ruoslahti E (2000). Aminopeptidase N is a receptor for tumor-homing peptides and a target for inhibiting angiogenesis. Cancer Res.

[45] Antczak C, De Meester I, Bauvois B (2001). Transmembrane proteases as disease markers and targets for therapy. J Biol Regul Homeost Agents.

[46] Hashida H, Takabayashi A, Kanai M, Adachi M, Kondo K, Kohno N, Yamaoka Y, Miyake M (2002). Aminopeptidase N is involved in cell motility and angiogenesis: its clinical significance in human colon cancer. Gastroenterology.

[47] Scott AM, Wolchok JD, Old LJ (2012). Antibody therapy of cancer. Nat Rev Cancer.

[48] Mentzel S, Assmann KJ, Dijkman HB, de Jong AS, van Son JP, Wetzels JF, Koene RA (1996). Inhibition of aminopeptidase A activity causes an acute albuminuria in mice: an angiotensin II-mediated effect?. Nephrol Dial Transplant.

[49] Reaux A, Iturrioz X, Vazeux G, Fournie-Zaluski MC, David C, Roques BP, Corvol P, Llorens-Cortes C (2000). Aminopeptidase A, which generates one of the main effector peptides of the brain renin-angiotensin system, angiotensin III, has a key role in central control of arterial blood pressure. Biochem Soc Trans.

[50] Liu ZQ, Mahmood T, Yang PC (2014). Western blot: technique, theory and trouble shooting. N Am J Med Sci.

[51] Edge SB, Compton CC (2010). The American Joint Committee on Cancer: the 7th edition of the AJCC cancer staging manual and the future of TNM. Ann Surg Oncol.

[52] Jorissen RN, Gibbs P, Christie M, Prakash S, Lipton L, Desai J, Kerr D, Aaltonen LA, Arango D, Kruhoffer M, Orntoft TF, Andersen CL, Gruidl M (2009). Metastasis-Associated Gene Expression Changes Predict Poor Outcomes in Patients with Dukes Stage B and C Colorectal Cancer. Clin Cancer Res.

