# Supplementary material for: Aminopeptidase A initiates tumorigenesis and enhances tumor cell stemness via TWIST1 upregulation in colorectal cancer
Source: Oncotarget. 2017 Feb 3;8(13):21266–80. doi: 10.18632/oncotarget.15072 (PMC5400582; doi:10.18632/oncotarget.15072)
Supplement: Supplementary file 1 [file oncotarget-08-21266-s001.pdf]

Aminopeptidase A initiates tumorigenesis and enhances tumor cell stemness via TWIST1 upregulation in colorectal cancer

SUPPLEMENTARY DATA

SUPPLEMENTARY MATERIALS AND METHODS

Cell cultures

SW480 and SW620 cells were cultured in Leibovitz's L-15 Medium (GIBCO, Life Technologies, California, United States) in an incubator at 37ºC without 5% CO_2. HT29 cells were cultured in RPMI-1640 (GIBCO, Life Technologies), HCT116 cells were grown in McCoy's 5A Medium (Sigma-Aldrich), and human embryonic kidney 293T cells were cultured in Dulbecco's modified Eagle medium (DMEM; GIBCO, Life Technologies) at 37ºC with 5% CO_2. Mouse CT26 undifferentiated colon carcinoma cells (a gift from Prof. Chi-Hung Lin of National Yang-Ming University, Taiwan) were cultured in RPMI-1640 medium (GIBCO, Life Technologies) at 37ºC with 5% CO_2. All culture media were supplemented with 10% fetal bovine serum (GIBCO, Life Technologies) and 1% penicillin/streptomycin (GIBCO, Life Technologies).

Site-directed mutagenesis

pLAS3w.Ppuro-APA was used as a template to modify mutagenesis nucleotides by *Pfu* DNA polymerase (Thermo Fisher Scientific) for 18 PCR cycles. Two primers were used for this process: forward 5'- GAGCATAGTGGCCACCGCCCATGAACCAA CAGATGC-3' and reverse 5'- GCATCTGTTGGTT CATGGGCGGTGGCCACTATGCTC-3'. To cleave methylated DNA, 1 µl DpnI enzyme (10 U/µl; Thermo Fisher Scientific) was added to the amplified product and held for 2 h at 37ºC. Following transformation in Escherichia coli DH10B (Sigma-Aldrich), transformants were selected for further sequencing to identify the D221A mutant.

Lentivirus production and infection

Knockdown experiments entailed lentivectors expressing shRNAs against human APA (shAPA) and TWIST1 (shTWIST#1, shTWIST#2), with luciferase (shLuc) serving as a negative control (all purchased from National RNAi Core Facility, Taiwan). ID numbers were TRCN0000051687 for the shAPA clone, TRCN0000378353 for shTwist#1, and TRCN0000378362 for shTwist#2. The shAPA clone sequence was 5'-CCGGGCTCAAGGACACGAACC TTATCTCGAGATAAGGTTCGTGTCCTTGAGCTT TTTG-3', the shTWIST#1 sequence 5'-CCGGAGTC CGCAGTCTTACGAGGAGCTCGAGCTCCTCGTA AGACTGCGGACTTTTTTG-3', and the shTWIST#2 sequence 5'-CCGGATGGCAAGCTGCAGCTATGTGC TCGAGCACATAGCTGCAGCTTGCCATTTTTTG-3'. HEK293T cells were used for lentivirus production according to National RNAi Core Facility protocols (http://rnai.genmed.sinica.edu.tw). Briefly, cells (4×10^5) were infected with virus supernatant (1:1 virus to medium) in the presence of 8 mg/ml protamine sulfate (Sigma-Aldrich) and held for 24 h at 37ºC. Viruses were removed prior to the addition of fresh medium containing puromycin (2.5 µg/ml; Sigma-Aldrich) or G418 (600 µg/ml; Life Technologies). Stable cells were selected after 7 days and transferred to fresh puromycin-containing medium.

Human colon tumor tissue microarray immunohistochemistry

Paraffin sections were baked at 65°C for 20 min and rehydrated with xylene, ethanol, and distilled water. Antigen retrieval was performed by heating tissue sections in 0.01 M citrate retrieval buffer (pH 6.0) in a pressure boiler for 30 min at 99°C (1 atm). Slides were cooled to room temperature and blocked with endogenous peroxidase in 3% H_2O_2 for 20 min. Next, slides were washed five times with 1% PBST and incubated at 4°C overnight with anti-BP1 (ab36122) or anti-TWIST (sc-15393) primary antibodies. After 10 washes with 1% PBST, slides were held at RT for 30 min with mouse/rabbit secondary antibodies (EnVision™ Detection Systems Peroxidase/DAB; Dako, Agilent Technologies, CA, USA). DAB staining was performed according to manufacturer instructions. Nuclei were counterstained with hematoxylin. Tissues were dehydrated with xylene, and water-based mounting medium was added to coverslips.

ALDH activity assays

We added 5 µl of the ALDH activity inhibitor diethylaminobenzaldehyde (DEAB) to a new test

tube. HT29 cells (1x10^6) were resuspended in 1 ml ALDEFLUOR™ Assay Buffer with 5 µl BODIPYTM-aminoacetaldehyde (BAAA) substrate (STEMCELL Technologies), mixed, and separated into two groups. One group (0.5 mL) was transferred to the DEAB tube, and both groups were incubated at 37ºC for 45 min, centrifuged, and resuspended in fresh assay buffer (0.5 mL). $ALDH^+$ populations were analyzed using a Cytomics FC 500 Series flow cytometry system (Beckman Coulter, Indianapolis, USA). The DEAB-treated group was used as a negative control for background levels.

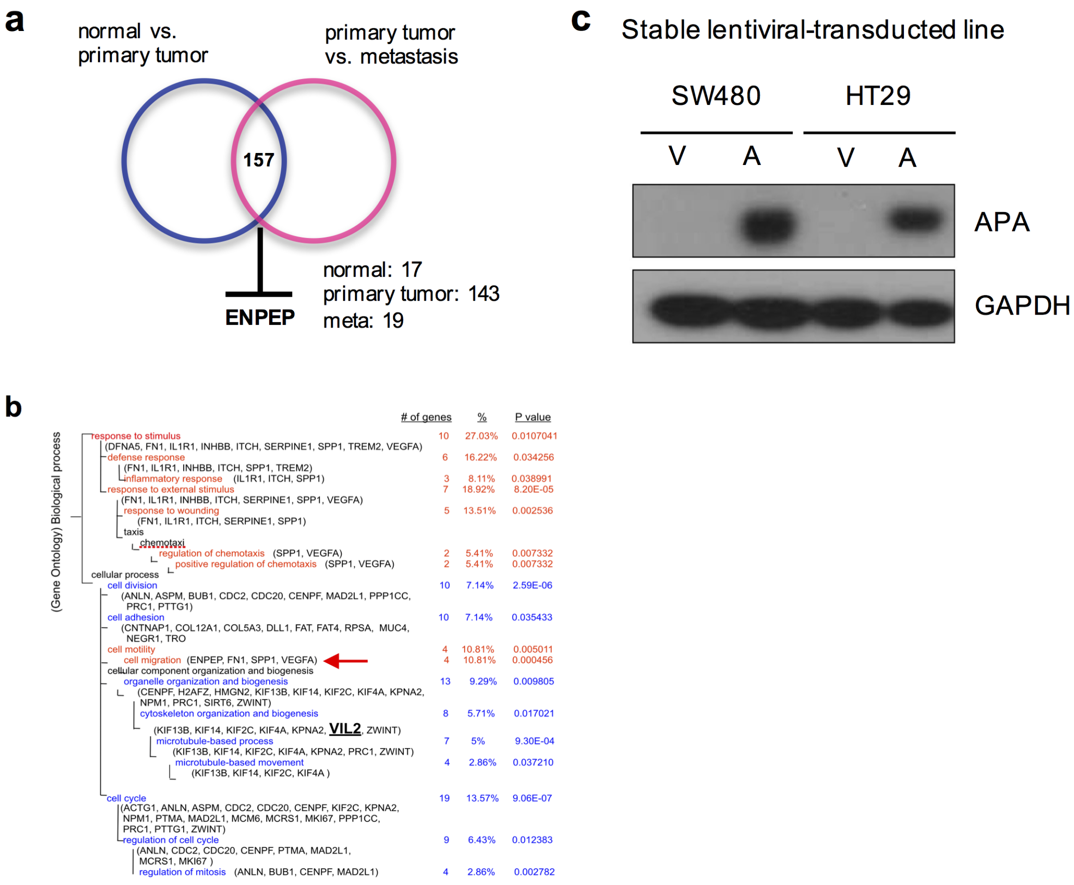

Supplmentary Figure 1: Confirmation of aminopeptidase A function in CRC. **a.** Blue, Venn diagram of overlapping genes differentially expressed in CRC normal (*n*=17) and primary tumor (*n*=147) tissue. Pink, differentially expressed genes between primary tumor and metastatic (*n*=19) tissue samples from CRC patients. **b.** Gene ontology chart showing the association between cell motility and the *ENPEP* gene (*P* <0.05). **c.** APA-overexpressing SW480 and HT29 cells were produced by infecting lentiviruses containing control or APA expression vectors. Cell extracts were used to confirm APA expression by Western blot.

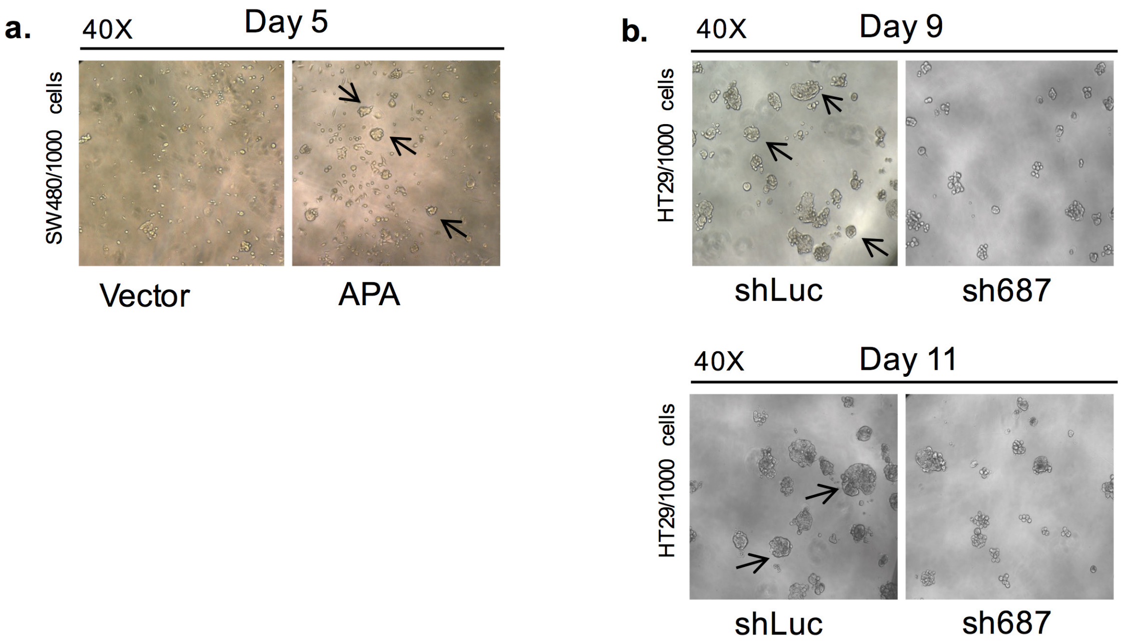

Supplmentary Figure 2: Sphere formation analyses for validating APA stemness function. a. Day 5 spheres per 1 000 stable APA-SW480 cells. **b.** Day 9 and day 11 spheres per 1 000 HT29-shLuc and shAPA cells.

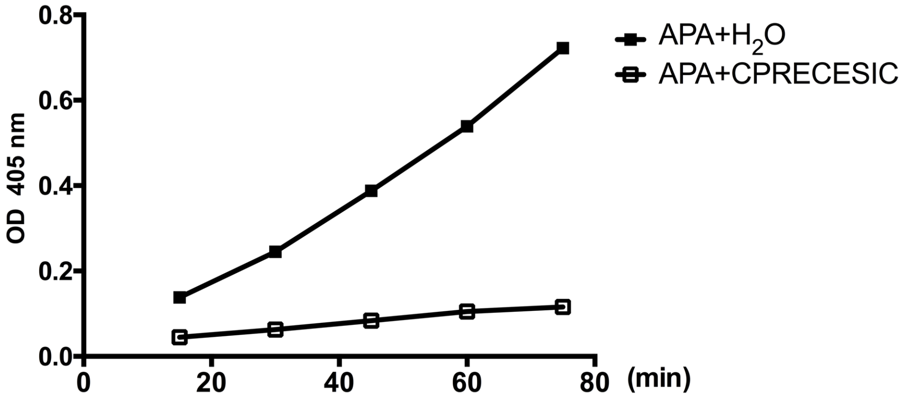

Supplmentary Figure 3: Confirmation of the inhibition of APA activity by CPRECESIC. Shown are OD 405 nm values for determining APA activity in APA-overexpressing SW480 cells following treatment with either APA + H_2O or APA + CPRECESIC peptide.

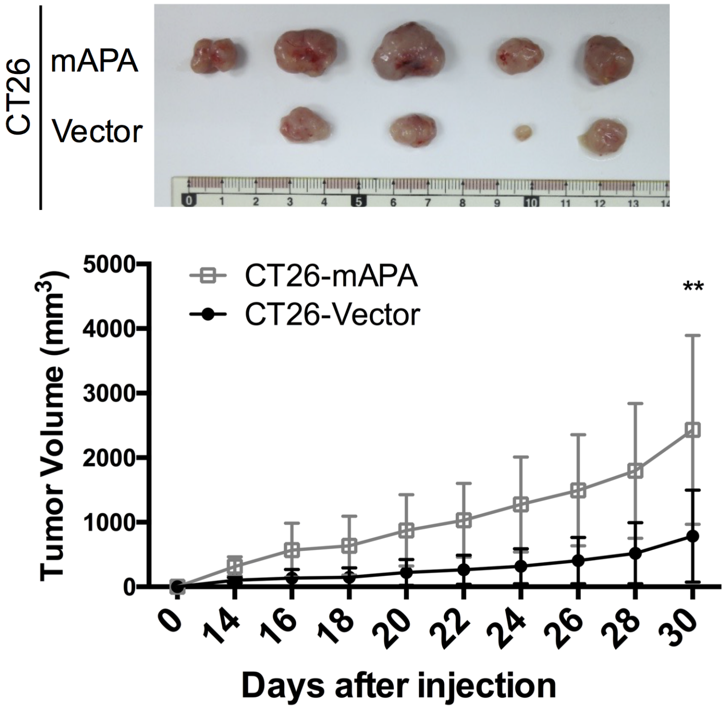

Supplmentary Figure 4: Overexpression of mouse APA in CT26 cells enhances tumorigenicity *in vivo*. CT26 cells expressing APA were injected subcutaneously into nude mice (n=5). Tumor volumes were measured once every 2 days.

Supplementary Table 1: Gender analysis of CRC patients in terms of APA or TWIST expression levels (IHC)

	Total (n=44)	APA Expression		*P*-value	TWIST Expression		*P*-value
		Negative (n=22)	Positive (n=22)		Negative (n=24)	Positive (n=20)	
Male	35 (79.5%)	17 (38.6%)	18 (40.9%)	0.500	20 (45.5%)	4 (9.1%)	0.378
Female	9 (20.5%)	5 (11.4%)	4 (9.1%)		15 (34.1%)	5 (11.4%)	

Supplementary Table 2: APA/TWIST co-expression levels (IHC) for colorectal cancer patients according to gender

APA Expression	TWIST Expression	Gender n (%)		*P*-value
		male	female	
Negative (n=17)	Neg (n=18)	14 (63.6)	4 (18.2)	0.675
	Pos (n=4)	3 (13.6)	1 (4.5)	
Positive (n=5)	Neg (n=6)	6 (27.3)	0 (0)	0.378
	Pos (n=16)	12 (54.5)	4 (18.2)	

Supplementary Table 3: List of qPCR human primers used in this study

Gene	Forward Primer (5’ - 3’)	Reverse Primer (5’ - 3’)
hCDH1	CCTGGGACTCCACCTACAGA	TGGATTCCAGAAACGGAGGC
hcMYC	GCCACGTCTCCACACATCAG	TCTTGGCAGCAGGATAGTCCTT
hENPEP	TGACACCGTTCACGTTAAGCA	GGAAGAGGCAAGTAGGCTACCA
hGPADH	TGGTTCACACCCATGACGAA	GGAGTCCACTGGCGTCTTCA
hNANOG	CAACCAGACCCAGAACATCC	TTCCAAGGCAGCCTCCAAG
hOCT4	ACCGAGTGAGAGGCAACC	TGAGAAAGGAGACCCAGCAG
hSLUG	TGTTGCAGTGAGGGCAAGAA	CAATGGCATGGGGGTCTGAAAG
hSOX2	CGAGTGGAAACTTTTGTCGGA	TGTGCAGCGCTCGCAG
hTWIST1	GGGAGTCCGcAGTCTTACGA	AGACCGAGAAGGCGTAGCTG
hVIM	GCAGGAGGCAGAAGAATGGT	CCACTTCACAGGTGAGGGAC

Supplementary Table 4: Antibodies used in this study

Protein	Antibody
APA	GTX102838, GeneTex, Inc.
GAPDH	MAB374, Merck Millipore Inc.
lamia A	MA3-1000, Thermo Fisher Scientific Inc.
NF-κB	GTX107678, GeneTex, Inc.
SLUG	GTX121924, GeneTex, Inc.
TWIST	GTX127310, GeneTex, Inc.
vimentin	GTX100619, GeneTex, Inc.
α-tubulin	GTX628802, GeneTex, Inc.
β-actin	MA5-15739, Thermo Fisher Scientific Inc.

APA, Aminopeptidase A; GAPDH, glyceraldehyde-3-phosphate dehydrogenase; NF-κB (nuclear factor kappa-light-chain-enhancer of activated B cells)

Supplementary Table 5: Clinicopathologic characteristics of patients that validation of APA and TWIST co-expression levels (Figure 6C and 6D) in colorectal cancer tissue array

See Supplementary File 1

Supplementary Table 6: Clinicopathologic characteristics of patients that APA expression related to prognosis (Figure 6E) in colorectal cancer tissue array

See Supplementary File 2
