# Supplementary material for: Aminopeptidase A initiates tumorigenesis and enhances tumor cell stemness via TWIST1 upregulation in colorectal cancer
Source: Oncotarget. 2017 Feb 3;8(13):21266–80. doi: 10.18632/oncotarget.15072 (PMC5400582; doi:10.18632/oncotarget.15072)
Supplement: Supplementary file 2 [file oncotarget-08-21266-s002.docx]

**Supplementary Table 5. Clinicopathologic characteristics of patients that validation of APA and TWIST co-expression levels (Figure 6C and 6D) in colorectal cancer tissue array.**

| Case | Age | Sex | Pathology diagnosis | Dukes’ Classification | T  (is-4) | N  (0-3) | M  (0 or 1) | APA | TWIST |
| --- | --- | --- | --- | --- | --- | --- | --- | --- | --- |
| 1 | 80 | M | adenocarcinoma | B2 | 3 | 0 | 0 | Low | Low |
| 2 | 75 | M | adenocarcinoma | D | 3 | 0 | 1 | Low | Low |
| 3 | 80 | M | adenocarcinoma | C1 | 3 | 1 | 0 | High | Low |
| 4 | 89 | M | adenocarcinoma | C2 | 3 | 1 | 0 | High | Low |
| 5 | 73 | M | adenocarcinoma | C2 | 3 | 3 | 0 | High | Low |
| 6 | 62 | F | others | D | 4 | 2 | 1 | Low | Low |
| 7 | 73 | M | mucinous carcinoma | C2 | 3 | 1 | 0 | Low | Low |
| 8 | 65 | F | adenocarcinoma | B2 | 3 | 0 | 0 | High | Low |
| 9 | 77 | M | adenocarcinoma | B2 | 3 | 0 | 0 | Low | Low |
| 10 | 69 | M | adenocarcinoma | D | 3 | 1 | 1 | Low | Low |
| 11 | 54 | F | adenocarcinoma | C2 | 4 | 3 | 0 | High | Low |
| 12 | 76 | M | adenocarcinoma | B2 | 3 | 0 | 0 | Low | Low |
| 13 | 55 | M | adenocarcinoma | B2 | 3 | 0 | 0 | Low | Low |
| 14 | 74 | M | adenocarcinoma | B2 | 3 | 0 | 0 | Low | Low |
| 15 | 78 | M | adenocarcinoma | B2 | 3 | 0 | 0 | High | Low |
| 16 | 55 | F | adenocarcinoma | B2 | 3 | 0 | 0 | High | High |
| 17 | 61 | F | adenocarcinoma | C1 | 2 | 1 | 0 | High | High |
| 18 | 76 | M | adenocarcinoma | D | 3 | 2 | 1 | High | High |
| 19 | 73 | M | adenocarcinoma | C2 | 2 | 0 | 0 | Low | High |
| 20 | 22 | M | adenocarcinoma | C2 | 3 | 2 | 0 | High | Low |
| 21 | 93 | M | adenocarcinoma | IS | IS | 0 | 0 | High | Low |
| 22 | 50 | M | adenocarcinoma | B2 | 4 | 0 | 0 | High | Low |
| 23 | 40 | F | adenocarcinoma | B2 | 3 | 0 | 0 | High | High |
| 24 | 54 | M | signet cell carcinoma | B2 | 3 | 0 | 0 | High | High |
| 25 | 73 | M | adenocarcinoma | C2 | 3 | 1 | 0 | Low | High |
| 26 | 79 | M | adenocarcinoma | A | 1 | 0 | 0 | High | High |
| 27 | 70 | M | adenocarcinoma | C2 | 3 | 3 | 0 | High | High |
| 28 | 86 | M | adenocarcinoma | B2 | 4 | 0 | 0 | High | High |
| 29 | 75 | M | adenocarcinoma | B2 | 3 | 0 | 0 | High | High |
| 30 | 67 | M | adenocarcinoma | B2 | 3 | 0 | 0 | Low | High |
| 31 | 60 | M | adenocarcinoma | B2 | 3 | 0 | 0 | Low | High |
| 32 | 67 | F | adenocarcinoma | D | 3 | 1 | 1 | Low | High |
| 33 | 73 | F | adenocarcinoma | IS | IS | 0 | 0 | High | High |
| 34 | 73 | M | adenocarcinoma | B1 | 2 | 0 | 0 | Low | Low |
| 35 | 64 | M | adenocarcinoma | C2 | 3 | 1 | 0 | High | Low |
| 36 | 80 | M | adenocarcinoma | B2 | 3 | 0 | 0 | Low | Low |
| 37 | 72 | M | adenocarcinoma | C2 | 3 | 3 | 0 | Low | Low |
| 38 | 81 | M | adenocarcinoma | C2 | 4 | 2 | 0 | Low | Low |
| 39 | 77 | M | adenocarcinoma | B2 | 3 | 0 | 0 | Low | Low |
| 40 | Unknown | M | Unknown | Unknown | Unknown | Unknown | Unknown | Low | High |
| 41 | Unknown | M | Unknown | Unknown | Unknown | Unknown | Unknown | Low | High |
| 42 | Unknown | M | Unknown | Unknown | Unknown | Unknown | Unknown | Low | Low |
| 43 | Unknown | M | Unknown | Unknown | Unknown | Unknown | Unknown | Low | Low |
| 44 | Unknown | M | Unknown | Unknown | Unknown | Unknown | Unknown | Low | Low |
| 45 | Unknown | M | Unknown | Unknown | Unknown | Unknown | Unknown | Low | Low |
| 46 | Unknown | M | Unknown | Unknown | Unknown | Unknown | Unknown | Low | High |
| 47 | Unknown | F | Unknown | Unknown | Unknown | Unknown | Unknown | Low | High |
| 48 | Unknown | F | Unknown | Unknown | Unknown | Unknown | Unknown | Low | High |
| 49 | Unknown | M | Unknown | Unknown | Unknown | Unknown | Unknown | Low | High |
| 50 | Unknown | M | Unknown | Unknown | Unknown | Unknown | Unknown | Low | High |
| 51 | Unknown | M | Unknown | Unknown | Unknown | Unknown | Unknown | High | High |
| 52 | Unknown | M | Unknown | Unknown | Unknown | Unknown | Unknown | High | High |
| 53 | Unknown | M | Unknown | Unknown | Unknown | Unknown | Unknown | High | High |
| 54 | Unknown | M | Unknown | Unknown | Unknown | Unknown | Unknown | High | Low |
| 55 | Unknown | M | Unknown | Unknown | Unknown | Unknown | Unknown | High | Low |
| 56 | Unknown | M | Unknown | Unknown | Unknown | Unknown | Unknown | High | Low |
| 57 | Unknown | M | Unknown | Unknown | Unknown | Unknown | Unknown | High | Low |
| 58 | Unknown | F | Unknown | Unknown | Unknown | Unknown | Unknown | High | Low |
| 59 | Unknown | M | Unknown | Unknown | Unknown | Unknown | Unknown | High | Low |
| 60 | Unknown | F | Unknown | Unknown | Unknown | Unknown | Unknown | Low | Low |
| 61 | Unknown | M | Unknown | Unknown | Unknown | Unknown | Unknown | High | Low |
| 62 | 82 | M | adenocarcinoma | B1 | 2 | 0 | 0 | High | High |
| 63 | 76 | M | adenocarcinoma | D | 4 | 2 | 1 | High | High |
| 64 | 81 | M | adenocarcinoma | D | 3 | 1 | 1 | High | High |
| 65 | 38 | M | adenocarcinoma | D | 3 | 2 | 1 | High | High |
| 66 | 75 | M | adenocarcinoma | B1 | 2 | 0 | 0 | High | Low |

M, Male; F, Female; T (is-4), Primary Tumor; N (0-3), Regional Lymph Nodes; M (0 or 1), Distant Metastases; APA, expression of APA; TWIST, expression of TWIST; IS, Carcinoma in situ
