# Supplementary material for: Aminopeptidase A initiates tumorigenesis and enhances tumor cell stemness via TWIST1 upregulation in colorectal cancer
Source: Oncotarget. 2017 Feb 3;8(13):21266–80. doi: 10.18632/oncotarget.15072 (PMC5400582; doi:10.18632/oncotarget.15072)
Supplement: Supplementary file 3 [file oncotarget-08-21266-s003.docx]

**Supplementary Table 6. Clinicopathologic characteristics of patients that APA expression related to prognosis (Figure 6E) in colorectal cancer tissue array.**

| **Case** | **Age** | **Sex** | **Pathology diagnosis** | **Dukes’ Classification** | **T**  **(is-4)** | **N**  **(0-3)** | **M**  **(0 or 1)** | **APA** |
| --- | --- | --- | --- | --- | --- | --- | --- | --- |
| 1 | 81 | M | adenocarcinoma | B2 | 3 | 0 | 0 | Low |
| 2 | 56 | M | signet cell carcinoma | C2 | 3 | 1 | 0 | Low |
| 3 | 75 | M | adenocarcinoma | C2 | 3 | 1 | 0 | High |
| 4 | 86 | F | adenocarcinoma | D | 3 | 2 | 1 | Low |
| 5 | 80 | M | adenocarcinoma | D | 3 | 0 | 1 | Low |
| 6 | 79 | M | adenocarcinoma | B2 | 3 | 0 | 0 | High |
| 7 | 63 | F | adenocarcinoma | D | 3 | 3 | 1 | High |
| 8 | 78 | F | adenocarcinoma | C2 | 3 | 1 | 0 | High |
| 9 | 63 | F | adenocarcinoma | C2 | 3 | 2 | 0 | Low |
| 10 | 72 | M | adenocarcinoma | B2 | 3 | 0 | 0 | Low |
| 11 | 74 | M | adenocarcinoma | D | 4 | 3 | 1 | High |
| 12 | 53 | M | adenocarcinoma | B1 | 2 | 0 | 0 | Low |
| 13 | 76 | M | adenocarcinoma | C2 | 3 | 1 | 0 | High |
| 14 | 60 | F | adenocarcinoma | D | 3 | 2 | 1 | High |
| 15 | 79 | M | adenocarcinoma | B1 | 2 | 0 | 0 | High |
| 16 | 64 | F | adenocarcinoma | C2 | 4 | 2 | 0 | High |
| 17 | 54 | M | adenocarcinoma | C2 | 3 | 1 | 0 | High |
| 18 | 55 | F | adenocarcinoma | D | 3 | 1 | 1 | High |
| 19 | 78 | M | adenocarcinoma | B1 | 2 | 0 | 0 | High |
| 20 | 68 | F | adenocarcinoma | B2 | 3 | 0 | 0 | High |
| 21 | 73 | M | adenocarcinoma | D | 3 | 1 | 1 | High |
| 22 | 74 | M | adenocarcinoma | C2 | 3 | 1 | 0 | Low |
| 23 | 80 | F | adenocarcinoma | B1 | 2 | 0 | 0 | High |
| 24 | 70 | M | adenocarcinoma | B2 | 3 | 0 | 0 | High |
| 25 | 75 | M | adenocarcinoma | C1 | 2 | 1 | 0 | High |
| 26 | 84 | M | adenocarcinoma | C2 | 3 | 2 | 0 | High |
| 27 | 68 | F | adenocarcinoma | C2 | 3 | 2 | 0 | High |
| 28 | 82 | M | signet cell carcinoma | C2 | 3 | 2 | 0 | Low |
| 29 | 60 | F | adenocarcinoma | A | 1 | 0 | 0 | High |
| 30 | 84 | M | adenocarcinoma | D | 3 | 0 | 1 | High |
| 31 | 80 | M | adenocarcinoma | B2 | 3 | 0 | 0 | Low |
| 32 | 75 | M | adenocarcinoma | D | 3 | 0 | 1 | Low |
| 33 | 80 | M | adenocarcinoma | C1 | 3 | 1 | 0 | High |
| 34 | 89 | M | adenocarcinoma | C2 | 3 | 1 | 0 | High |
| 35 | 73 | M | adenocarcinoma | C2 | 3 | 3 | 0 | High |
| 36 | 62 | F | others | D | 4 | 2 | 1 | Low |
| 37 | 73 | M | mucinous carcinoma | C2 | 3 | 1 | 0 | Low |
| 38 | 65 | F | adenocarcinoma | B2 | 3 | 0 | 0 | High |
| 39 | 77 | M | adenocarcinoma | B2 | 3 | 0 | 0 | Low |
| 40 | 69 | M | adenocarcinoma | D | 3 | 1 | 1 | Low |
| 41 | 54 | F | adenocarcinoma | C2 | 4 | 3 | 0 | High |
| 42 | 76 | M | adenocarcinoma | B2 | 3 | 0 | 0 | Low |
| 43 | 82 | F | adenocarcinoma | B2 | 3 | 0 | 0 | High |
| 44 | 55 | M | adenocarcinoma | B2 | 3 | 0 | 0 | Low |
| 45 | 74 | M | adenocarcinoma | B2 | 3 | 0 | 0 | Low |
| 46 | 78 | M | adenocarcinoma | B2 | 3 | 0 | 0 | High |
| 47 | 55 | F | adenocarcinoma | B2 | 3 | 0 | 0 | High |
| 48 | 61 | F | adenocarcinoma | C1 | 2 | 1 | 0 | High |
| 49 | 76 | M | adenocarcinoma | D | 3 | 2 | 1 | High |
| 50 | 73 | M | adenocarcinoma | C2 | 2 | 0 | 0 | Low |
| 51 | 22 | M | adenocarcinoma | C2 | 3 | 2 | 0 | High |
| 52 | 93 | M | adenocarcinoma | IS | IS | 0 | 0 | High |
| 53 | 50 | M | adenocarcinoma | B2 | 4 | 0 | 0 | High |
| 54 | 40 | F | adenocarcinoma | B2 | 3 | 0 | 0 | High |
| 55 | 54 | M | signet cell carcinoma | B2 | 3 | 0 | 0 | High |
| 56 | 73 | M | adenocarcinoma | C2 | 3 | 1 | 0 | Low |
| 57 | 79 | M | adenocarcinoma | A | 1 | 0 | 0 | High |
| 58 | 70 | M | adenocarcinoma | C2 | 3 | 3 | 0 | High |
| 59 | 86 | M | adenocarcinoma | B2 | 4 | 0 | 0 | High |
| 60 | 75 | M | adenocarcinoma | B2 | 3 | 0 | 0 | High |
| 61 | 76 | M | adenocarcinoma | B1 | 3 | 0 | 0 | Low |
| 62 | 77 | M | adenocarcinoma | D | 4 | 2 | 1 | High |
| 63 | 78 | M | adenocarcinoma | B2 | 3 | 0 | 0 | High |
| 64 | 79 | F | adenocarcinoma | D | 3 | 0 | 1 | High |
| 65 | 85 | F | adenocarcinoma | C2 | 4 | 1 | 0 | Low |
| 66 | 65 | F | adenocarcinoma | D | 4 | 3 | 1 | High |
| 67 | 72 | F | adenocarcinoma | B1 | 2 | 0 | 0 | High |
| 68 | 86 | M | leiomyosarcoma | B2 | 4 | 0 | 0 | Low |
| 69 | 67 | M | adenocarcinoma | B2 | 3 | 0 | 0 | Low |
| 70 | 60 | M | adenocarcinoma | B2 | 3 | 0 | 0 | Low |
| 71 | 67 | F | adenocarcinoma | C2 | 3 | 1 | 0 | High |
| 72 | 65 | F | adenocarcinoma | B2 | 3 | 0 | 0 | High |
| 73 | 73 | F | adenocarcinoma | B2 | 4 | 0 | 0 | High |
| 74 | 73 | F | adenocarcinoma | D | 4 | 3 | 1 | High |
| 75 | 59 | F | adenocarcinoma | B2 | 4 | 0 | 0 | High |
| 76 | 75 | M | adenocarcinoma | B2 | 3 | 0 | 0 | High |
| 77 | 70 | F | adenocarcinoma | B2 | 3 | 0 | 0 | Low |
| 78 | 69 | F | adenocarcinoma | C2 | 3 | 1 | 0 | Low |
| 79 | 77 | M | adenocarcinoma | B2 | 4 | 0 | 0 | Low |
| 80 | 74 | F | adenocarcinoma | D | 3 | 2 | 1 | Low |
| 81 | 67 | F | mucinous carcinoma | B2 | 3 | 0 | 0 | High |
| 82 | 50 | M | adenocarcinoma | D | 3 | 0 | 1 | Low |
| 83 | 85 | M | adenocarcinoma | B2 | 4 | 0 | 0 | Low |
| 84 | 76 | M | adenocarcinoma | B2 | 3 | 0 | 0 | Low |
| 85 | 67 | F | adenocarcinoma | D | 3 | 1 | 1 | Low |
| 86 | 73 | F | adenocarcinoma | IS | IS | 0 | 0 | High |
| 87 | 73 | M | adenocarcinoma | B1 | 2 | 0 | 0 | Low |
| 88 | 86 | M | adenocarcinoma | B2 | 3 | 0 | 0 | Low |
| 89 | 64 | M | adenocarcinoma | C2 | 3 | 1 | 0 | High |
| 90 | 76 | M | adenocarcinoma | B1 | 2 | 0 | 0 | High |
| 91 | 81 | M | adenocarcinoma | C2 | 3 | 1 | 0 | Low |
| 92 | 89 | M | adenocarcinoma | C2 | 4 | 2 | 0 | Low |
| 93 | 81 | M | adenocarcinoma | B1 | 2 | 0 | 0 | Low |
| 94 | 81 | M | adenocarcinoma | B1 | 2 | 0 | 0 | High |
| 95 | 53 | F | adenocarcinoma | D | 4 | 0 | 1 | High |
| 96 | 50 | M | adenocarcinoma | B2 | 3 | 0 | 0 | Low |
| 97 | 80 | M | adenocarcinoma | B2 | 3 | 0 | 0 | High |
| 98 | 71 | M | adenocarcinoma | B1 | 3 | 0 | 0 | High |
| 99 | 56 | M | mucinous carcinoma | D | 4 | 0 | 1 | High |
| 100 | 67 | F | adenocarcinoma | C2 | 3 | 1 | 0 | High |
| 101 | 73 | M | adenocarcinoma | C2 | 3 | 2 | 0 | Low |
| 102 | 70 | M | adenocarcinoma | C2 | 4 | 2 | 0 | Low |
| 103 | 80 | M | adenocarcinoma | B2 | 3 | 0 | 0 | Low |
| 104 | 72 | M | adenocarcinoma | C2 | 3 | 3 | 0 | Low |
| 105 | 81 | M | adenocarcinoma | C2 | 4 | 2 | 0 | Low |

M, Male; F, Female; T (is-4), Primary Tumor; N (0-3), Regional Lymph Nodes; M (0 or 1), Distant Metastases; APA, expression of APA; IS, Carcinoma in situ
